# Role of biomarker SOCS1 in peritoneal dialysis-associated peritoneal fibrosis and immune infiltration based on machine learning screening

**DOI:** 10.3389/fphar.2025.1646948

**Published:** 2025-10-01

**Authors:** Xin Ma, Xiaona He, Yun Wang, Marcin Grzegorzek, Wenjie Long, Hongxi Chen, Fang Gao, Nan Mao, Xinyu Huang

**Affiliations:** ^1^ Department of Nephrology, The First Affiliated Hospital of Chengdu Medical College, Chengdu, China; ^2^ Department of Clinical Medicine, School of Clinical Medicine, Chengdu Medical College, Chengdu, China; ^3^ Sichuan Clinical Research Center for Geriatrics, The First Affiliated Hospital of Chengdu Medical College, Chengdu, China; ^4^ Institute of Medical Informatics, University of Luebeck, Luebeck, Germany; ^5^ German Research Center for Artificial Intelligence (DFKI), Luebeck, Germany; ^6^ ExpandAI GmbH, Luebeck, Germany

**Keywords:** SOCS-1, dialysis-associated peritoneal fibrosis, immune infiltration, machine learning, peritoneal dialysis

## Abstract

**Introduction/Objectives:**

Chronic peritoneal dialysis (PD) induces peritoneal fibrosis through bioincompatible fluid exposure and inflammation. This study aimed to identify fibrosis biomarkers via bioinformatics, assess immune cell correlations, and validate diagnostic utility in clinical/animal models.

**Methods:**

We analyzed the GSE125498 peritoneal fluid transcriptome to identify differentially expressed genes (DEGs). Functional enrichment, protein interaction networks, and pathway analyses were performed. Three machine learning algorithms screened diagnostic markers, validated by ROC curves and nomogram modeling. Immune infiltration patterns were correlated with biomarkers. Clinical validation included SOCS-1/TGF-β1 quantification in 86 PD patients’ effluent and a CKD rat fibrosis model to assess SOCS-1/TGF-β/Smad pathway interactions.

**Results:**

Four hub genes (PIM2, HSH2D, MYO3B, SOCS1; AUC = 0.914) were identified as PD fibrosis biomarkers. Monocyte infiltration increased significantly in long-term PD cohorts and inversely correlated with all biomarkers. SOCS1 exhibited positive associations with CD4+/CD8+ T cells and M1 macrophages, but negative correlations with resting mast cells and monocytes. Clinically, SOCS-1 levels in PD effluent showed non-linear temporal dynamics. Rat models confirmed SOCS-1 overexpression in fibrotic peritoneum and its functional link to TGF-β/Smad signaling.

**Conclusion:**

SOCS-1 emerges as a novel biomarker for PD-related peritoneal fibrosis, with monocyte-mediated mechanisms playing a critical role. Animal studies implicate SOCS-1 in TGF-β/Smad-driven fibrogenesis. These findings provide mechanistic insights and translational tools for monitoring PD complications.

## Introduction

End-stage renal disease (ESRD) represents the primary cause of mortality on a global scale, with approximately 11% of patients worldwide opting for peritoneal dialysis (PD) treatment (Liyanage et al., 2015; Cho et al., 2021; Teitelbaum, 2021). PD is an easily accepted and accessible treatment modality in some low-income and lower-middle-income countries owing to its simple and easy-to-grasp technique, low cost, and better preservation of residual renal function than hemodialysis (Cho et al., 2021). Some developed countries have advocated a PD-first policy (Li et al., 2017). However, prolonged PD causes changes in the morphology and function of the peritoneum, which leads to peritoneal fibrosis, with signs of fibrosis detected in 50%–80% of patients within 1 or 2 years after PD (Jagirdar et al., 2019). After peritoneal fibrosis occurs in PD patients, it affects the filtration of toxins and fluids in PD patients, leading to ultrafiltration failure and impaired solute clearance. Severe fibrosis can result in peritoneal adhesions or encapsulating peritoneal sclerosis, which can cause intestinal obstruction or difficulty in draining dialysate. This, in turn, increases the likelihood of recurrent peritonitis, and ultimately, the patient may be required to discontinue peritoneal dialysis (Smit et al., 2004; Moinuddin et al., 2015). There are two main causes of peritoneal fibrosis: the fibrotic process itself and inflammation caused by non-physiological components and infection. These two processes are usually bidirectional and interact (Zhou et al., 2016).

The features of peritoneal fibrosis, including the epithelial-to-mesenchymal transition (EMT) of mesothelial cells, fibroblast activation, extracellular matrix deposition, and angiogenesis, are well-documented (Zhou et al., 2016). Transforming growth factor-β1 (TGF-β1) is pivotal in activating peritoneal fibroblasts and inducing EMT mediated by dialysate, thereby playing a crucial role in peritoneal fibrosis pathogenesis (Yao et al., 2008; Padwal and Margetts, 2016). TGF-β regulates gene expression in fibrosis by inducing the phosphorylation of Smad2/3 proteins (Kolb et al., 2001; Loureiro et al., 2011; Helmke et al., 2019; Su et al., 2021).

Peritoneal fibrosis results from progressive changes in the peritoneum driven by inflammatory and infectious events, including the secretion of extracellular mediators and leukocyte recruitment. Numerous studies have underscored the role of immune cell infiltration in the development of peritoneal fibrosis (Terri et al., 2021). The peritoneum has a distinctive anatomical structure known as the papilla or fat-associated lymphoid cluster (FALC), which consists mainly of macrophages, mesothelial cells, and B-1 lymphocytes. FALCs play a role in the recruitment of polymorphonuclear leukocytes and monocytes during the first phase of inflammation and subsequent induction of adaptive immunity (Terri et al., 2021). Evaluating the differences in immune cell infiltration in the peritoneal effluent of patients undergoing long-duration PD compared with that of patients undergoing short-duration PD is crucial for understanding the immunological mechanisms of peritoneal fibrosis in PD. This will aid in the identification of potential immunotherapeutic targets. Diagnostic tools for peritoneal fibrosis in patients undergoing PD include biomarker testing of PD effluent, pathological testing of peritoneal tissue biopsies (considered the gold standard), and estimation of peritoneal fibrosis by peritoneal function. Substances used for the early diagnosis of peritoneal fibrosis in peritoneal effluent include CA125 (Ditsawanon et al., 2014; Barreto et al., 2015), interleukin (IL)-6 (Davies, 2014), plasminogen activator inhibitor 1 (Barreto et al., 2013; Lopes Barreto et al., 2015), CCL18 (Ahmad et al., 2010), effluent decoy receptor 2 appearance rate (Yang et al., 2022), and IL-17A (Rodrigues-Díez et al., 2014).

However, there is a lack of diagnostic markers based on sequencing big data acquisition. The aim of this study was to screen biomarkers of peritoneal fibrosis using a bioinformatics approach by analysing a microarray dataset related to peritoneal fluid from patients undergoing PD obtained from the Gene Expression Omnibus (GEO) database to identify differentially expressed genes (DEGs). Following DEG screening, Gene Ontology (GO), Kyoto Encyclopedia of Genes and Genomes (KEGG), gene set enrichment analysis (GSEA), and protein-protein interaction (PPI) network analysis were conducted to identify diagnostic markers for the development of peritoneal fibrosis in patients undergoing PD using various machine learning algorithms. The CIBERSORT method was used to analyse the differences in immune cell subpopulation infiltration between patients undergoing long- and short-duration PD and investigate the relationship between diagnostic markers and infiltrating immune cells. Additionally, we explored the association between hub gene expression and peritoneal fibrosis in PD effluent from patients undergoing PD using clinical samples and assessed its predictive efficacy as a diagnostic marker for the development of peritoneal fibrosis. The relationship between hub genes and peritoneal fibrosis after PD was validated using a rat model of peritoneal fibrosis. We hope to search for new diagnostic markers and initially explore their role in peritoneal dialysis-associated peritoneal fibrosis using available sequencing data, clinical samples, and animal model.

## Materials and methods

### Data download and data preprocessing

In this study, the GSE125498 dataset was sourced from the GEO (http://www.ncbi.nlm.nih.gov/geo/) database, which contains 13 patients undergoing long-term and 20 patients undergoing short-term PD. Genotyping was performed on all subjects using the GPL10558 Illumina HumanHT-12 V4.0. After downloading the raw gene expression data, we performed a comprehensive bioinformatics analysis of the gene expression data using R (version 4.2.0).

### DEG identification

We used the ‘limma’ package to identify genes that are differently expressed between patients undergoing long-term and short-term PD. We used strict statistical significance criteria of *P* < 0.05 and |log_2_FC| > 1. This analysis revealed a set of genes with significant expression differences that reflected the biological variations associated with the duration of dialysis. To effectively visualise these findings, ggplot2 (Villanueva and Chen, 2019) and pheatmap packages were used to draw volcano and heat maps of the DEGs, respectively. (According to the introduction of GSE125498 data, short-term peritoneal dialysis is defined as PD duration of 0–24 months, while PD duration ≥25 months is defined as long-term peritoneal dialysis).

### Functional enrichment analysis

We used GO (Ashburner et al., 2000) and KEGG (Kanehisa, 2000) to understand the functions and pathways of DEGs. GO analysis consisted of three main parts: cellular component (CC), molecular function (MF), and biological process (BP), which helped us understand how genes work. The GOplot (Walter et al., 2015), clusterProfiler (Yu et al., 2012) and ggplot2 packages were used to perform enrichment analyses. We identified significantly enriched categories with an adjusted *P*-value of less than 0.05.

The STRING online database (http://www.string-db.org/) (Franceschini et al., 2012) was used to analyse and construct PPI networks for DEGs. Genes with a confidence score of 0.4 or above were selected to ensure the reliability of the network analysis. Visual representations of the interactions between the selected genes were created using Cytoscape (v3.7.2) (Smoot et al., 2011) to facilitate an understanding of the gene connections and identification of key regulatory hubs. The importance of DEGs was determined by the betweenness centrality (BC) (Mahmoody et al., 2016) score, which was calculated using the CytoNCA plugin. The MCODE plugin was used to filter the significant nodes (central proteins) within the PPI networks (Cline et al., 2007).

### GSEA

GSEA (Subramanian et al., 2007) was used to facilitate comparison between the groups of genes, each representing a distinct biological state. The gene sets were similar, allowing the assessment of their expression in conjunction with one another. The change in gene expression between the groups was calculated, and GSEA was conducted with 1000 permutations to obtain normalised enrichment scores (NESs). An adjusted *P*-value threshold of <0.05, in conjunction with the clusterProfiler package, was used to identify gene sets that were significantly enriched based on expression changes across different temporal contexts.

### Construction of disease prediction model and screening diagnostic markers

Machine learning (ML) algorithms are used to improve the behaviour of a system by utilising computer performance, analysing and mining data to obtain patterns, and making predictions about the data. This study used LM to identify pivotal gene features. The algorithms used were XGBoost (Zhong et al., 2018), Random Forest (RF), and Support Vector Machine (SVM). To ensure the reliability of the results, the samples from the GSE125498 dataset were divided into training and testing sets at a 1:1 ratio. We identified crucial marker genes that overlapped among the three algorithms.

The sensitivity and specificity of our predictive model were evaluated using receiver operating characteristic (ROC) curves (Heagerty et al., 2000). The area under the ROC curve (AUC) was calculated to demonstrate the capacity of the model to discriminate between positive and negative classes. The ‘pROC’ package was used to generate the ROC curve and assess model performance at varying threshold settings.

### Evaluation and analysis of immune cell infiltration and correlation analysis between immune cells

In this study, we used the CIBERSORT (https://cibersort.stanford.edu/) method, which is a technique for estimating the composition and abundance of immune cells based on gene expression data (Chen et al., 2018). The LM22 gene signature file was used to analyse peritoneal cells from patients undergoing dialysis, with only results with CIBERSORT *P*-values <0.05. The corrplot package (McKenna et al., 2016) was used to demonstrate the distribution of immune cells and their interrelationships. CIBERSORT effectively elucidates the immune cell types in patients undergoing dialysis.

To study the correlation between the immune score of characteristic immune cells and expression levels of diagnostic marker genes, Spearman’s rank correlation coefficient was calculated. The ggcorrplot (Wang et al., 2019) and ggplot2 packages were used for visualisation.

### Clinical specimens

Patients who underwent regular PD were included in this study. PD effluent and fasting serum samples were collected from 86 patients for 4 h of peritoneal equilibration experiments, excluding those with recent or recurrent peritonitis. Patients were divided into three groups based on PD duration: long-term dialysis (n = 53, PD ≥ 36 months), initial dialysis (n = 22, PD < 12 months), and intermediate-term dialysis (n = 11, 36 > PD ≥ 12 months). *SOCS1* and TGF-β1 expression in PD effluent was measured. Informed consent was obtained from all patients before collecting laparotomy effluent and serum samples. Clinical data collected included sex, age, dialysis duration, weekly Kt/V values, Ccr values, transit patterns, and ultrafiltration volumes. The study was approved by the Ethics Committee of the First Affiliated Hospital of Chengdu Medical College (approval number: 2022CYFYIRB-BA-APr01). The Watson formula was used to calculate peritoneal Kt/V in patients undergoing PD.

### ELISA

The collected PD effluent samples were centrifuged at 4 °C and 3,000 rpm in a low-speed centrifuge for 10 min. The supernatant was then frozen at −80 °C. The levels of *SOCS1* and TGF-β1 in PD effluent were determined using *SOCS1* and TGF-β1 ELISA reagent kits (Invitrogen, Carlsbad, CA, United States), respectively.

### Animals

In total, 24 adult male Sprague-Dawley rats (aged between 6 and 8 weeks, with a mean weight of 180–220 g) were purchased from Chengdu ShuoDa Laboratory Animal Company. The rats were randomly assigned to three different groups: a control group (n = 8), CKD group (n = 8), and CKD combined PD group (n = 8). Rats of the CKD and CKD combined PD groups received 2.5% adenine suspension (200 mg/kg/day) orally daily, whereas the control group received the same amount of saline orally. From day 15 of the experiment, rats of the CKD combined PD group received daily intraperitoneal injections of 4.25% high-glucose PD solution (100 mL/kg/d), whereas the control and CKD groups received the same number of saline injections.

Following the successful creation of the model, the following methods will be used to assess the peritoneal function of the rats: Serum and peritoneal transudate were collected from rats, and both the initial and 4-h post-transudate samples were analyzed using biochemical assay kits for urea nitrogen and glucose. The urea nitrogen concentration (D) in the 4-h post peritoneal transudate and the corresponding serum urea nitrogen concentration (P), as well as the glucose concentrations in the 4-h post peritoneal transudate (D4) and initial transudate (D0), were determined. Ultrafiltration volume (UF), mass transport glucose (MTG), dialysate urea nitrogen/serum urea nitrogen ratio (D/P), and D4/ D ratios were calculated according to established equation to evaluate peritoneal function in the rats. [MTG (nmol/kg) = (glucose concentration at the beginning of PD × the initial volume of injected dialysate) − (glucose concentration after PD × the final volume of reserved dialysate)].

On day 42 of the experiment, we euthanised the rats with phenobarbital and retained the specimens for further analysis. All procedures strictly adhered to the ethical standards approved by the Experimental Animal Ethics Committee of Chengdu Medical College.

### Histological study of the peritoneum

The thickness and degree of fibrosis of the peritoneal wall layer were assessed using haematoxylin and eosin-stained tissue sections and Masson’s trichrome stain (200×). Semi-quantitative analyses were conducted using Image-ProPlus (version 6.0) to determine the peritoneal thickness and degree of fibrosis in each section, with an average of five independent measurements. Immunohistochemical staining involved incubation with primary antibodies against a-SMA (Affinity, AF1032), type I collagen (Affinity, AF7001), or *SOCS1* (Enogene, E20-74362). The results were visualised under a light microscope (200×), and a semi-quantitative analysis of the positively stained area was performed using Image-ProPlus 6.0.

### Western blot analysis

Proteins were extracted from the membranes and denatured before electrophoresis. The membranes were incubated at 4 °C overnight with antibodies specific to TGF-*β*1 (Affinity, AF1027), Smad2/3 (Affinity, AF6367), and phosphorylated Smad2/3 (Affinity, AF3367). Horseradish peroxidase-labelled secondary antibodies were applied to the membranes for 1 h to facilitate visualisation. Immunoreactive proteins were detected using an enhanced chemiluminescence system (Amersham Biosciences, United Kingdom) with high sensitivity and clarity.

### Statistical analysis

Data are presented as means ± standard deviation (SD) to provide a comprehensive overview of the sample population. To ascertain whether the observed differences between the groups were significant, one-way ANOVA was used. This technique compares the mean values of multiple groups. Non-linearity was assessed using restricted cubic splines in the R package ‘rms’. A *P* value of less than 0.05 indicated a significant difference, which is a commonly accepted threshold for biomedical research.

## Results

### Identification of DEGs

**FIGURE 1:**
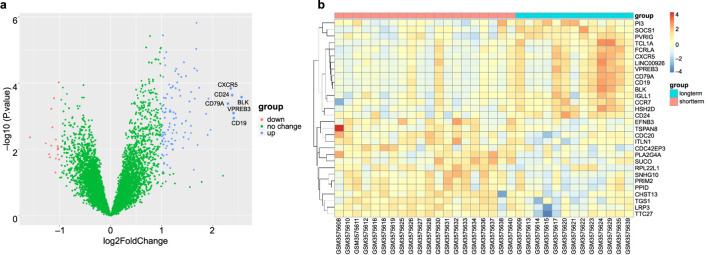
Differential expression analysis. **(a)** Volcano plot of GSE125498 between long-term peritoneal dialysis (PD) group and short-term PD group. Each dot in the volcano map represents a gene. **(b)** Heatmap of the top 30 differentially expressed genes (DEGs) that shows differences in gene expression between groups.

A comparative analysis was conducted to examine the effects of long- and short-term PD, utilising data from the GSE125498 dataset. This analysis yielded 121 DEGs. In total, 103 genes were significantly upregulated, and 18 genes were significantly downregulated in the long-term dialysis group. A volcano plot was constructed to illustrate the differences between the two patient groups (Figure 1a). Additionally, a heat map was generated to show the 15 most upregulated and 15 most downregulated genes (Figure 1b). These findings demonstrate that the length of PD affects gene expression.

### Functional enrichment analysis

**FIGURE 2:**
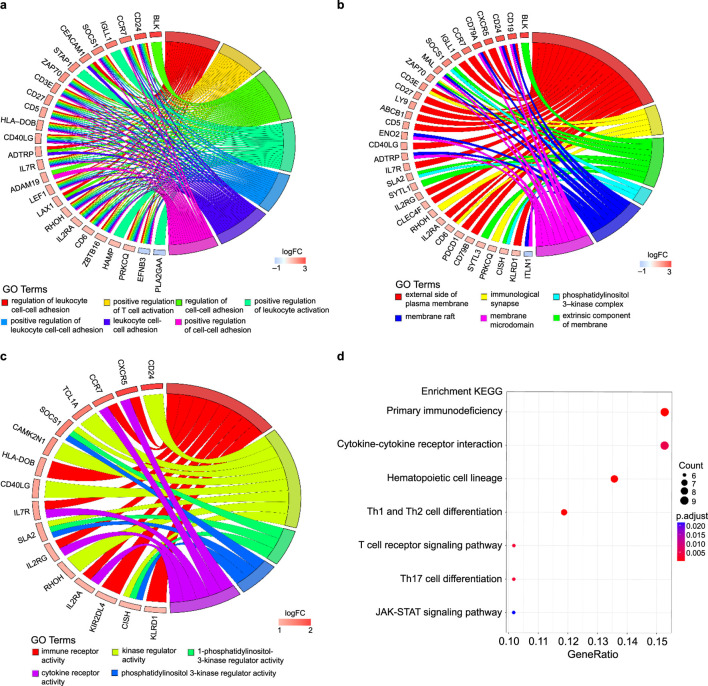
Enrichment analyses of differentially expressed genes (DEGs). **(a–c)** Circos plot to indicate the relationship between genes and GO terms (a for BP, b for CC, and c for MF). **(d)** KEGG pathway enrichment analyses. The horizontal axis represents the GeneRatio of the pathway and the vertical axis represents the pathway list.

In total, 179 GO terms were identified in our analysis; 168 terms were identified as related to BP, six to CC, and five to MF. The Benjamini–Hochberg method was used to ascertain the significance of these terms, with a threshold of *P* < 0.05. The DEGs were enriched in BPs linked to inflammation, including the regulation of leukocyte cell-cell adhesion, positive regulation of T cell activation, regulation of cell-cell adhesion, positive regulation of leukocyte activation, and positive regulation of leukocyte cell-cell adhesion; CCs of the external side of the plasma membrane, immunological synapses, extrinsic components of the membrane, the phosphatidylinositol 3-kinase complex, and membrane rafts; and MFs of immune receptor activity, kinase regulator activity, and 1-phosphatidylinositol-3-kinase regulator activity (Figures 2a–c). Using a *P*-value of less than 0.05, the KEGG pathways linked to the DEGs are presented in Figure 2d. The related pathways were mainly involved in primary immunodeficiency, cytokine-cytokine receptor interactions, hematopoietic cell lineages, Th1 and Th2 cell differentiation, and the T cell receptor signalling pathway. The GOplot, clusterProfiler, and ggplot2 packages in R were used to analyse and visualise the biological classification of DEGs.

**FIGURE 3:**
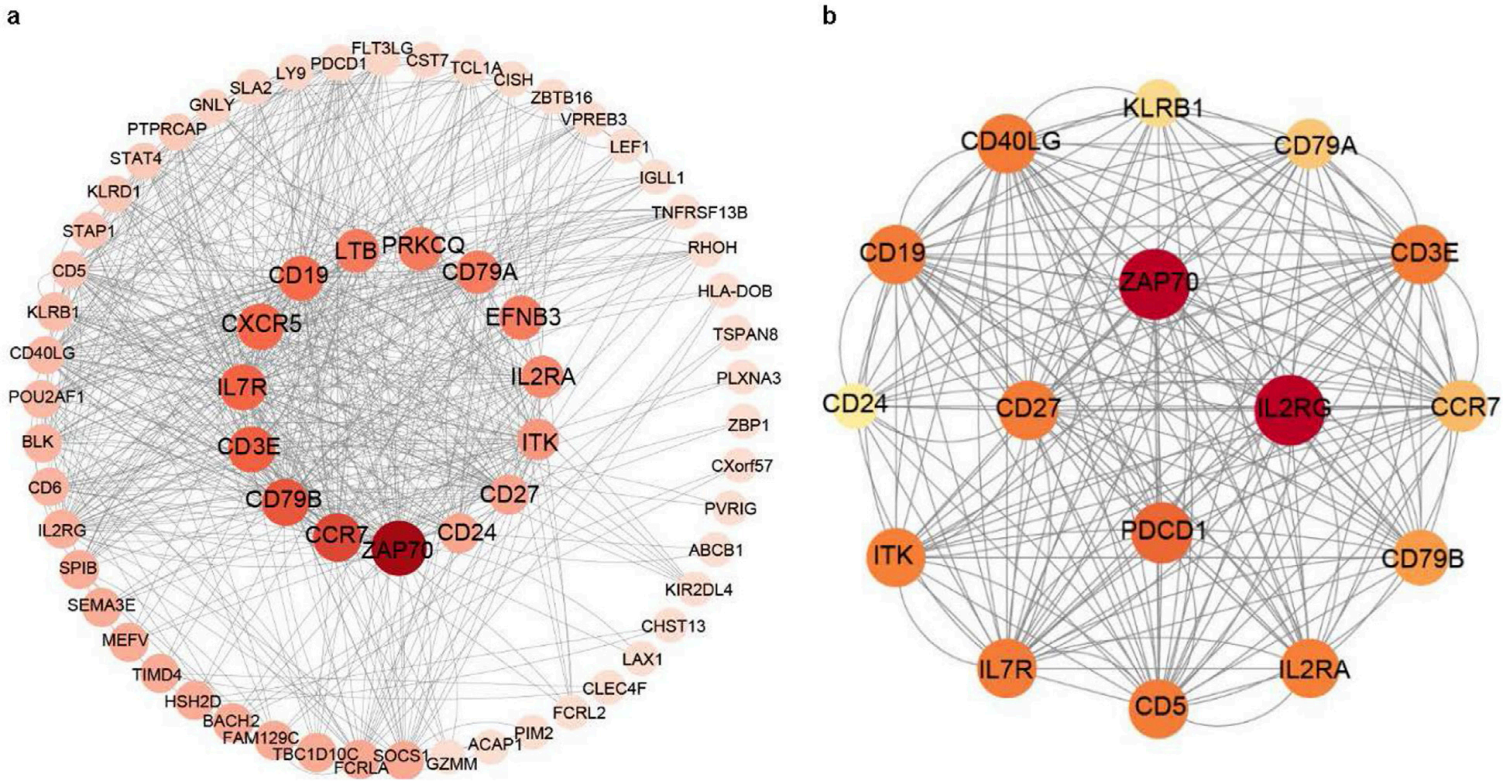
PPI networks of the DEGs and module analysis. **(a)** PPI network of DEGs in patients with long-term peritoneal dialysis compared with short-term. Genes were sorted by BC score, which was calculated using the CytoNCA plug-in. The darkest coloured gene had the highest BC score. **(b)** One of the most important modules was identified using the MCODE plug-in of Cytoscape in the PPI network.

The PPI network data file from STRING was imported into Cytoscape, and a PPI network of DEGs was constructed (Figure 3a). We used the CytoNCA plug-in to calculate the BC score for each gene, sorted according to the BC score, and finally filtered the top five genes: the zeta chain of T cell receptor-associated protein kinase 70 (*ZAP70*), C-C motif chemokine receptor 7 (*CCR7*), CD79b molecule (*CD79B*), CD3 epsilon subunit of T cell receptor complex (*CD3E*), and IL-7 receptor (*IL7R*). One of the most important modules was recognised using the MCODE plug-in in Cytoscape. Sixteen central genes were identified (Figure 3b), including *ZAP70*, *IL2RG*, *PDCD1*, *CD27*, and *IL7R* as the top five genes.

### Screening and verification of diagnostic markers based on ML

The screened DEGs were used to construct prediction models, and the samples of the original dataset were randomly divided into training and validation sets according to a 1:1 ratio. Three ML methods were used to construct prediction models on this grouping.

**FIGURE 4:**
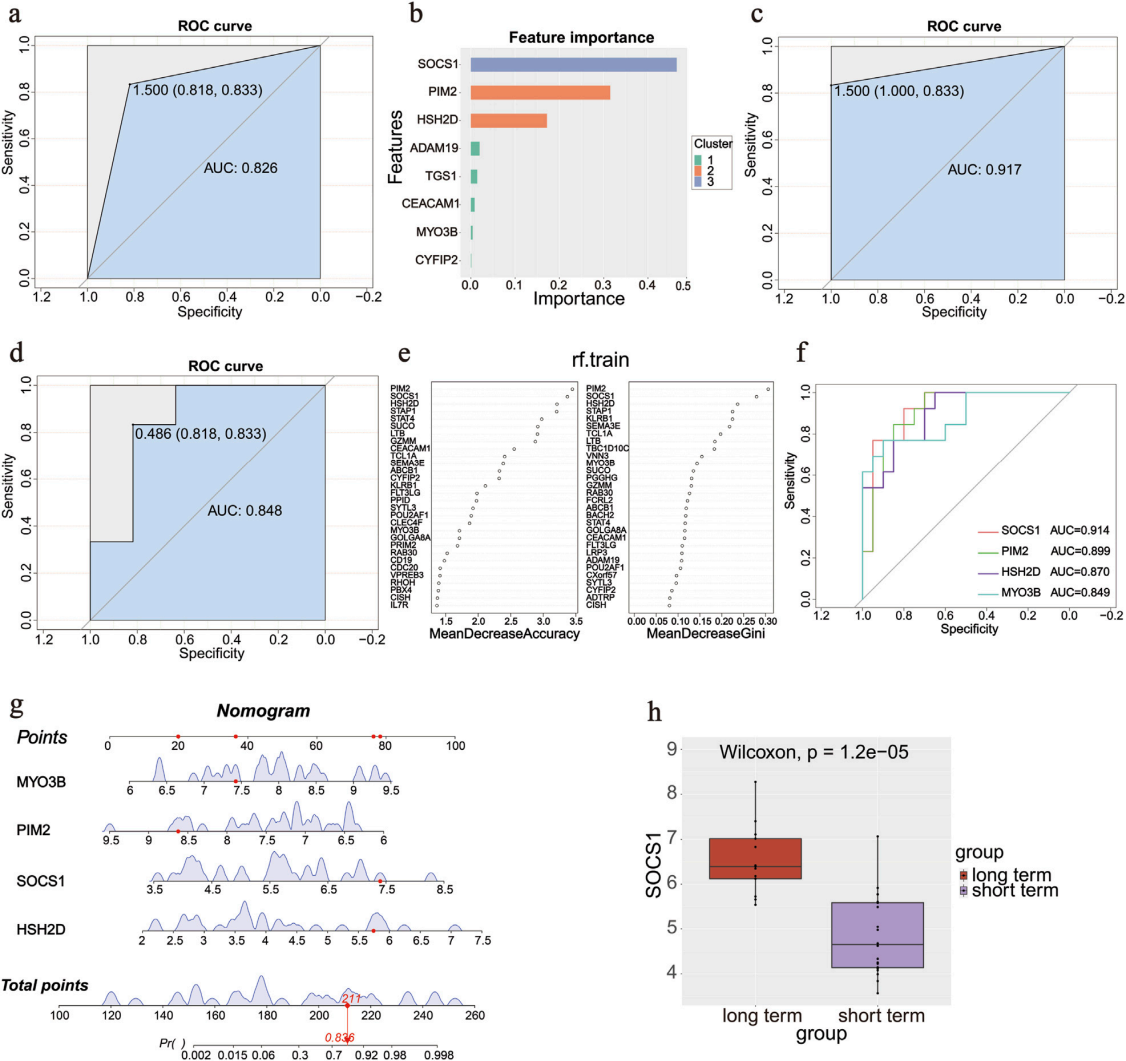
Diagnostic gene screening process. **(a)** ROC curve in the validation set of the XGBoost machine learning method. **(b)** Variable importance of genes in the XGBoost machine learning method. **(c)** ROC curve of seven screened genes by rfe function in the SVM algorithm. **(d)** ROC curve in the validation set in the random forest method. **(e)** Gini coefficients of the genes in the random forest method. **(f)** ROC curves of four screened genes using three methods. **(g)** Nomogram of four screened genes to predict the occurrence of an outcome. **(h)** Box plot of *SOCS1* between groups.

The prediction models were first constructed in the training set using the XGBoost ML method and validated in the validation set. The ROC curve is shown in Figure 4a, with an AUC of 0.826 (sensitivity: 0.833, specificity: 0.818). The variable importance was calculated (Figure 4b); the top values were obtained for *SOCS1*, *PIM2*, and *HSH2D*. We continued to use the SVM algorithm to construct a model to screen important genes. Characteristic genes were screened using the rfe function of the caret package in R: *FLT3LG*, *MYO3B*, *PIM2*, *SOCS1*, *CD40LG*, *PBX4*, and *HSH2D*. A prediction model was constructed using the seven screened genes (Figure 4c). Finally, the prediction model was constructed using an RF, and the ROC curve was plotted in the validation set with an AUC of 0.848 (sensitivity: 0.833, specificity: 0.818; Figure 4d). The Gini coefficients for these genes are shown in Figure 4e. The genes at the top of the three prediction models, *SOCS1*, *PIM2*, *HSH2D*, and *MYO3B*, were taken as intersection sets. Single gene ROC curves were plotted (Figure 4f), among which *SOCS1* had the largest AUC value (AUC = 0.914). The Wilcoxon test was used to assess the variability of the four genes between the groups and generate box plots (Figures 4g,h).

### Immune infiltration

**FIGURE 5:**
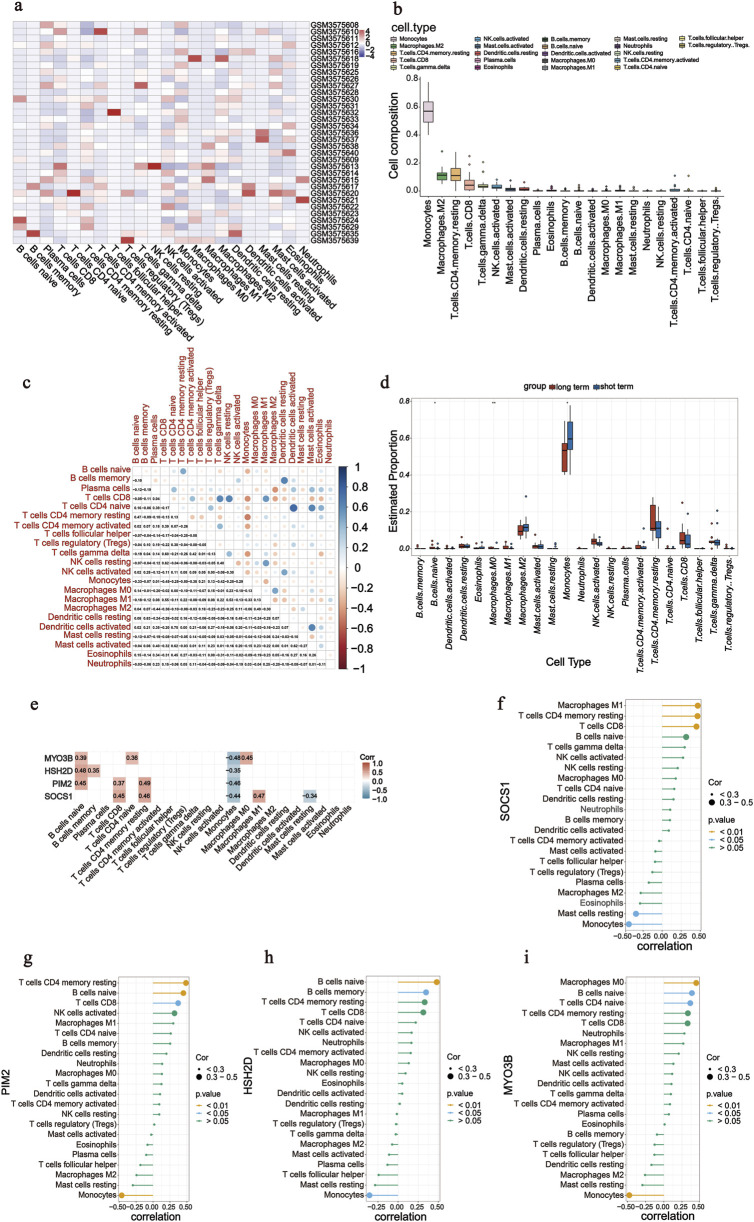
Analysis of immune cell infiltration. **(a)** Heatmap of 22 immune cells in GSE125498. **(b)** Box plot of overall expression of 22 immune cells in all patients. **(c)** Correlation of 22 immune cells in peritoneal dialysate effluent from peritoneal dialysis patients was evaluated. Dot colour indicates if they are positively related (blue) or negatively related (red). **(d)** Box diagram shows the proportion of 22 groups of immune cells in two groups. **(e–i)** Correlation was calculated between four screened genes (*SOCS1*, *PIM2*, *HSH2D*, and *MYO3B*) and 22 immune cells.

The percentage of infiltrating immune cells was analysed using Cibersort; monocytes were most prevalent, followed by M2 macrophages and memory/resting CD4 T cells (Figures 5a,b). The correlation between 22 groups of immune cells in patients undergoing long-term PD was evaluated (Figures 5a,b). Activated dendritic cells and naïve CD4 T cells had a significant positive correlation (*r* = 0.75). Memory/resting CD4 T cells and monocytes had a negative correlation (*r* = −0.50). A comparative analysis of immune cell infiltration in the two groups showed significant differences in three types of cells: naïve B cells, M0 macrophages, and monocytes (Figures 5c,d). Monocyte counts were significantly lower in patients undergoing long-term PD than in those undergoing short-term PD. In contrast, the number of naïve B cells and M0 macrophages increased, confirming the significance of reducing these cell types in the long-term PD immune microenvironment. We analysed the correlation between the screened significant genes and immune cells and calculated their Spearman’s correlation coefficients. Monocytes were negative for four of the screened genes (Figure 5e). *SOCS1* showed a positive correlation with CD8 T cells (*r* = 0.45, *P* = 0.008), memory/resting CD4 T cells (*r* = 0.46, *P* = 0.007), and M1 macrophages (*r* = 0.47, *P* = 0.006) but negative correlation with resting mast cells (*r* = −0.34, *P* = 0.050) and monocytes (*r* = −0.44, *P* = 0.012; Figure 5f).

### Single-gene GSEA analysis

**FIGURE 6:**
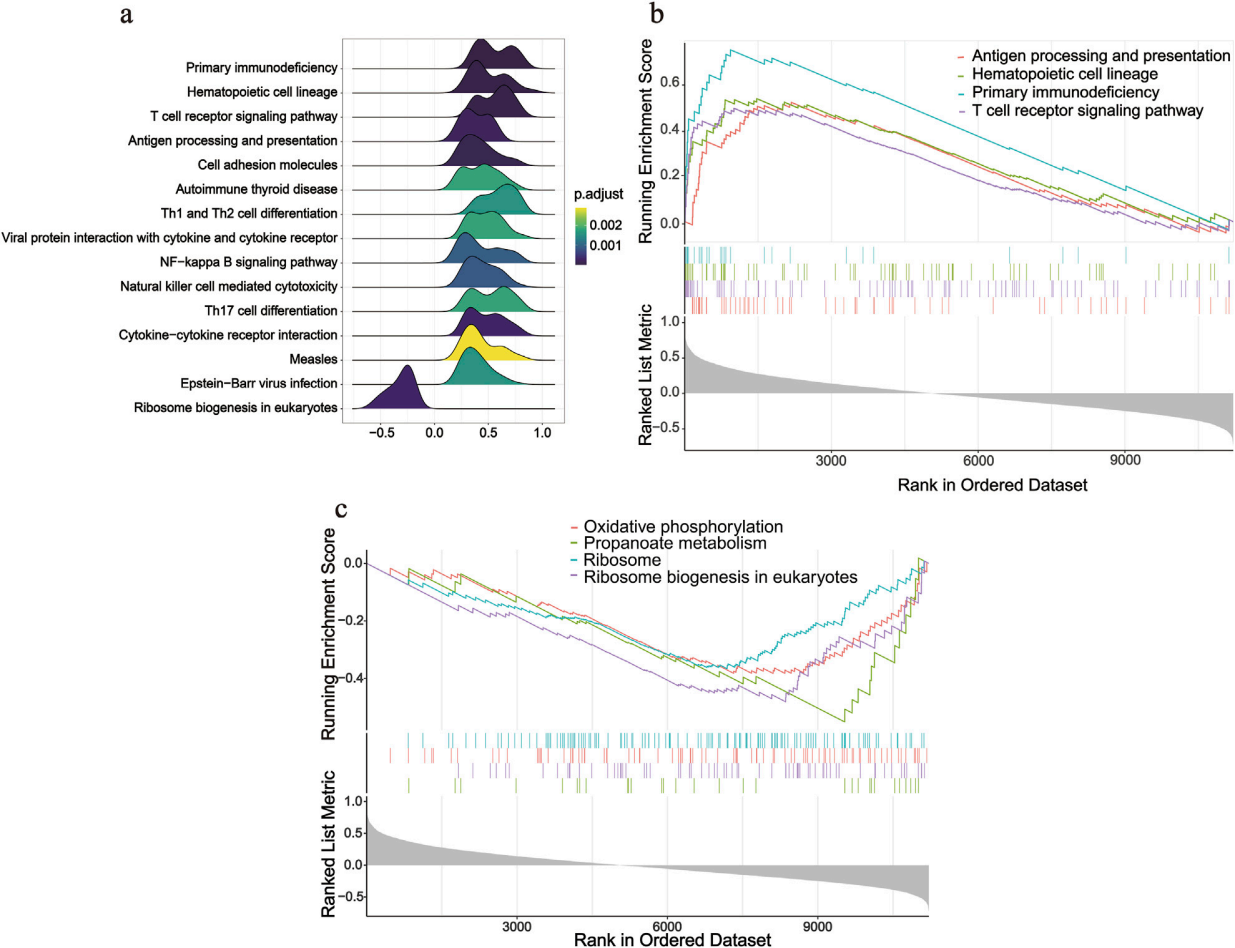
Single-gene GSEA analysis of *SOCS1*. **(a)** Ridgeline plot of top 15 pathways according to NES. **(b)** Enriched KEGG pathways positively correlated with *SOCS1*. **(c)** Enriched KEGG pathways negatively correlated with *SOCS1*.

After screening *SOCS1*, we conducted single-gene GSEA to investigate its involvement in peritoneal fibrosis progression. Using this method, we identified 33 signalling pathways linked to peritoneal fibrosis in patients undergoing PD, with 15 pathways specifically highlighted (Figure 6a). Several pathways, including primary immunodeficiency, hematopoietic cell lineage, T cell receptor signalling pathway, and antigen processing and presentation, were upregulated in the high *SOCS1* expression group (Figure 6b). NES was the main statistical parameter used to test gene set enrichment results. Patients with primary immunodeficiency had the highest NES value. Additionally, several downregulated pathways included ribosome biogenesis in eukaryotes, propanoate metabolism, ribosomes, and oxidative phosphorylation (Figure 6c). Among the downregulated pathways, eukaryotic ribosome biogenesis displayed the highest NES value.

### Level of *SOCS1* expression in PD effluent was significantly correlated with dialysis time

**FIGURE 7:**
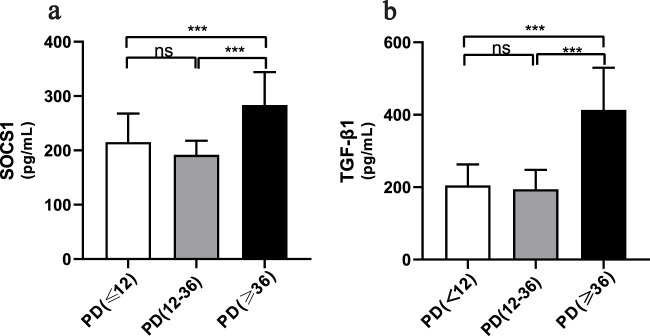
*SOCS1* levels in peritoneal dialysis patients at different periods of time. **(a)** Levels of *SOCS1* in peritoneal fluid effluent of patients in different treatment groups. **(b)** Levels of TGF-β1 in peritoneal fluid effluent of patients in different treatment groups. *p < 0.05 vs. Control; **p < 0.01 vs. Control; ***p < 0.001 vs. Control.

**FIGURE 8:**
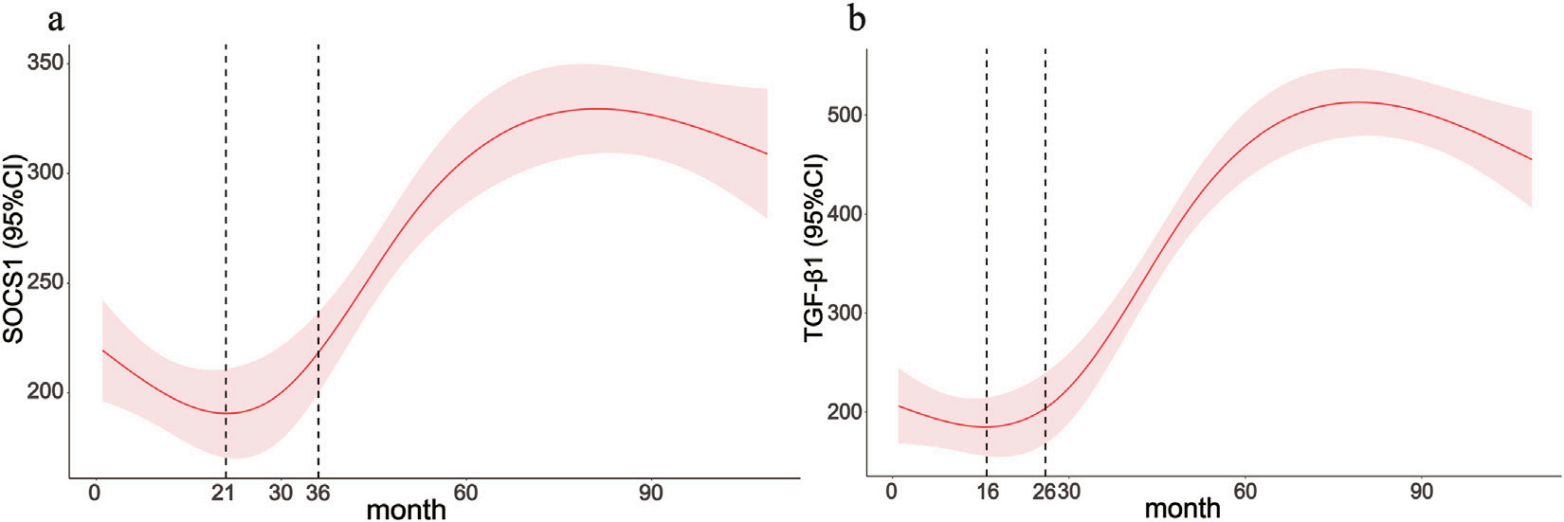
Clinical application value of the *SOCS1* gene. **(a,b)** Associations between the peritoneal dialysis time with *SOCS1* and TGF-β1 were assessed by restricted cubic spline curves based on a linear model estimation using ordinary least squares.

**FIGURE 9:**
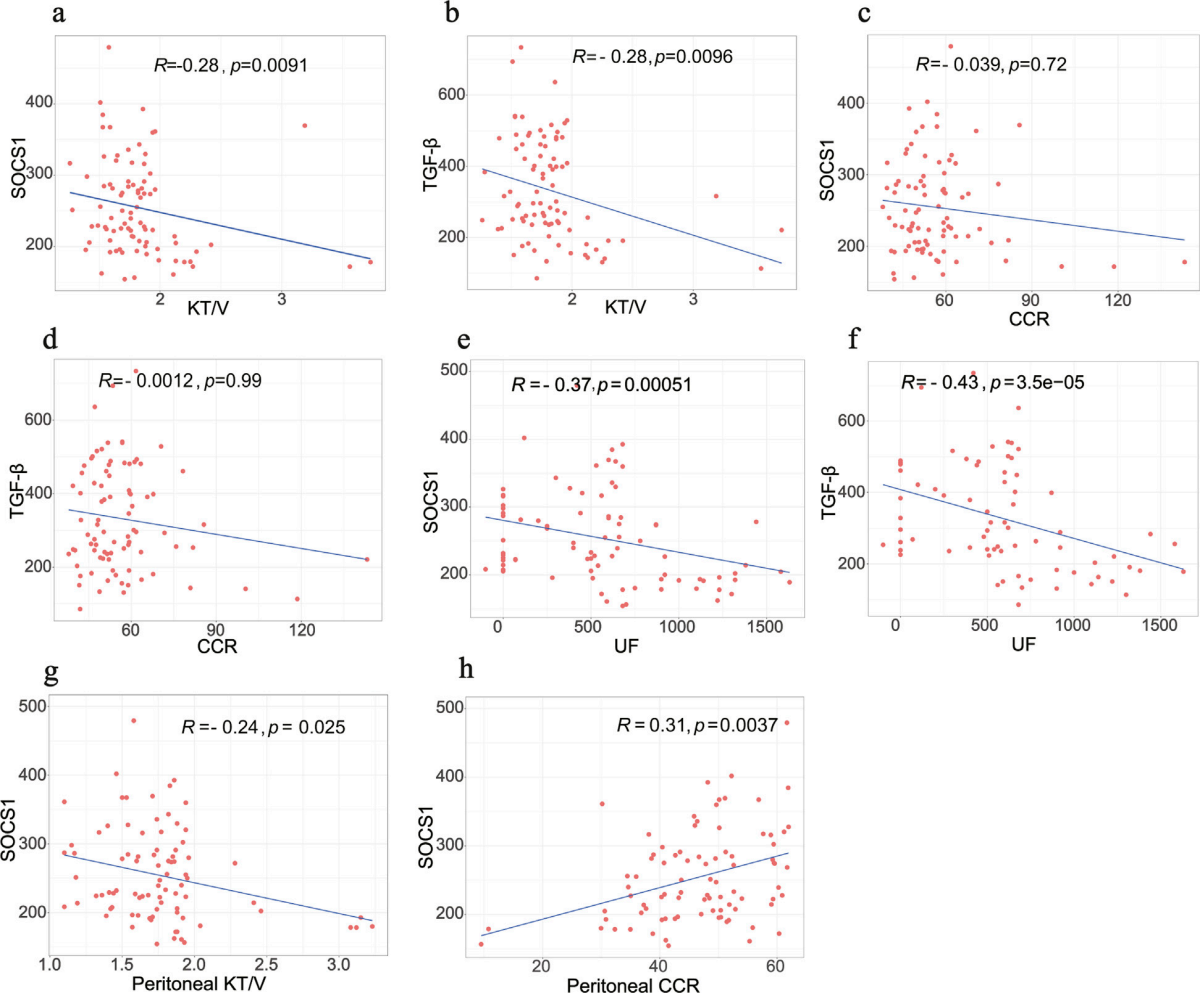
Correlation analysis between biomarkers and PET data in clinical samples. **(a,b)** Correlation between SOCS1 and TGF-β1 with total KT/V, respectively. **(c,d)** Correlation between SOCS1 and TGF-β1 with total CCR, respectively. **(e,f)** Correlation between SOCS1 and TGF-β1 with UF, respectively. **(g,h)** Correlation between peritoneal KT/V and peritoneal CCR with SOCS1, respectively.

The average dialysis durations for the three patient groups were 4.00 months (1.00–5.25), 28 months (23.00–30.00), and 54.00 months (43.00–87.00), respectively (Table 1). Laboratory testing of all samples was conducted. *SOCS1* levels were significantly higher in the long-term peritoneal group than in the initial peritoneal group (*P <* 0.05; Figure 7a). However, no significant difference in *SOCS1* level was observed between patients in the intermediate-term peritoneal group and initial peritoneal group (*P* > 0.05; Figure 7a). To evaluate the predictive efficacy of *SOCS1* for peritoneal fibrosis progression, we assessed TGF-β1 expression in the three groups. TGF-β1 levels were significantly higher in the peritoneal dialysate of patients undergoing long-term dialysis than in that of those in the initial PD group (*P* < 0.05; Figure 7b). Consistent with the results of SOCS1, there was no significant difference in TGF-β1 level between patients in the intermediate-term peritoneal group and initial peritoneal group (*P* > 0.05; Figure 7b). Restricted cubic splines indicated an increase in *SOCS1* expression in the PD fluid with prolonged dialysis duration after 21 months (Figure 8a). The expression of TGF-β1 in the PD effluent exhibited an upward trend with dialysis duration, particularly noticeable after 16 months of dialysis (Figure 8b). Therefore, TGF-β1 may be an early predictor of peritoneal fibrosis onset. We then analysed the correlations between SOCS1 and TGF-β1 and various clinical indicators in PET. The results showed that SOCS1 and TGF-β1 were negatively correlated with total KT/V, with correlation coefficients of −0.28 (Figures 9a,b). However, the correlation analysis results between the two and total CCR were not statistically significant (Figures 9c,d). For UF, SOCS1 and TGF-β1 were found to be inversely correlated with UF, with correlation coefficients of −0.37 and −0.43, respectively (Figures 9e,f). With regard to peritoneal-related indicators, the results indicated that SOCS1 exhibited a negative correlation with peritoneal KT/V (Figure 9g) and a positive correlation with peritoneal CCR (Figure 9h). These findings indicate that TGF-β1 appears earlier than SOCS1 in the peritoneal effluent of PD patients and demonstrates better correlation in some PET data. However, they also suggest that SOCS1 may become a new predictor of the development of peritoneal fibrosis in patients undergoing PD.

**TABLE 1:** General clinical measurements and the baseline of patients undergoing PD in three groups.

Variables	PD < 12 group (*n* = 22)	36 > PD ≥ 12 group (*n* = 11)	PD ≥ 36 group (*n* = 53)	*p*-value
Age (year)	45.73 ± 10.00	51.09 ± 14.21	50.34 ± 12.57	0.290
Sex (male/female)	9/13	6/5	20/33	0.241
BMI (kg/m^2^)	23.22 ± 1.82	23.39 ± 3.37	24.42 ± 3.14	0.205
PD duration (month) (*M*, *P*25, *P*75)	4.00 (1.00, 5.25)	28.00 (23.00, 30.00)	54.00 (43.00, 87.00)	<0.001
Urine volume (mL)	700 (425, 1225)	300 (200, 1000)	0 (0, 350)	<0.001
Ultrafiltration volume	946.8 ± 72.11	718.2 ± 136.1	364.9 ± 38.32	<0.001
Dialysis parameters				
Peritoneal Kt/V (weekly)	1.93 (1.59, 3.17)	1.69 (0.88, 1.88)	1.74 (1.46, 1.86)	<0.05
Renal Kt/V (weekly)	0.30 (0.05, 0.94)	0.19 (0.01, 1.39)	0.00 (0.01, 0.09)	<0.001
Total Kt/V (weekly)	2.03 ± 0.68	1.92 ± 0.23	1.75 ± 0.20	<0.05
Peritoneal Ccr (L/week/1.73 m^2^)	38.63 ± 7.19	44.09 ± 19.29	50.41 ± 8.22	<0.001
Renal Ccr (L/week/1.73 m^2^)	9.24 (1.66, 32.14)	3.64 (0.39, 39.99)	0.00 (0.00, 4.53)	<0.001
Total Ccr (L/week/1.73 m^2^)	55.14 (41.94, 61.78)	51.77 (48.86, 58.96)	52.74 (48.43, 60.64)	0.863

PD, peritoneal dialysis; BMI, body mass index; Kt/V, urea removal index. Data are expressed as mean ± SD.

### *SOCS1* is strongly associated with peritoneal fibrosis

**FIGURE 10:**
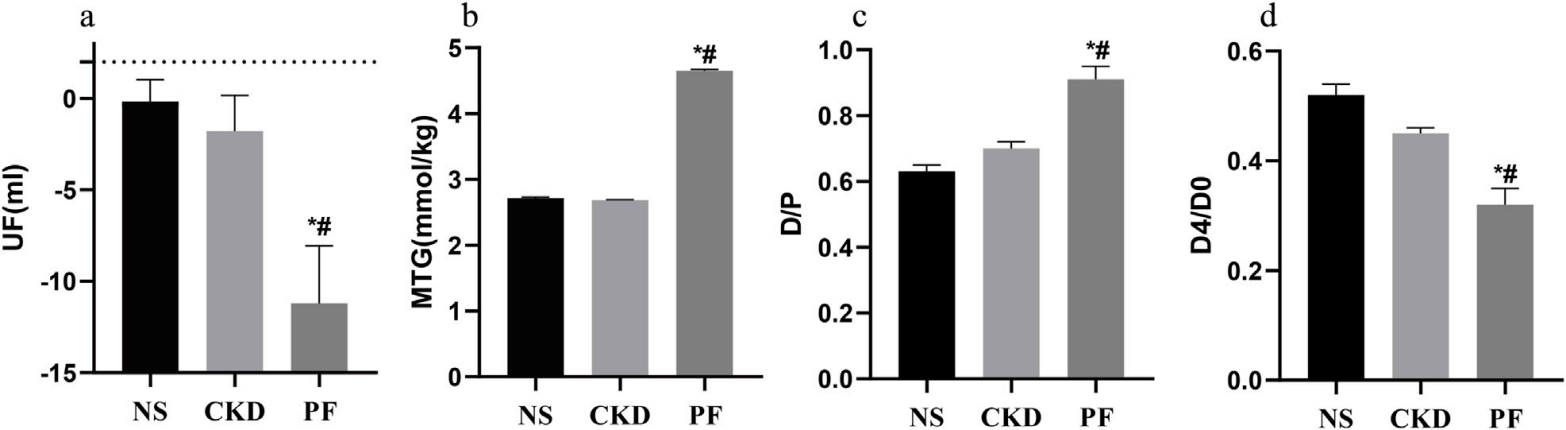
Peritoneal function-related parameters of rats in each group. **(a)** Parameters related to UF in every group. **(b)** Parameters related to MTG in every group. **(c)** Parameters related to D/P in every group. **(d)** Parameters related to D4/D0 in every group. *p < 0.05 vs. NS; #*p* < 0.05 vs. CKD.

**FIGURE 11:**
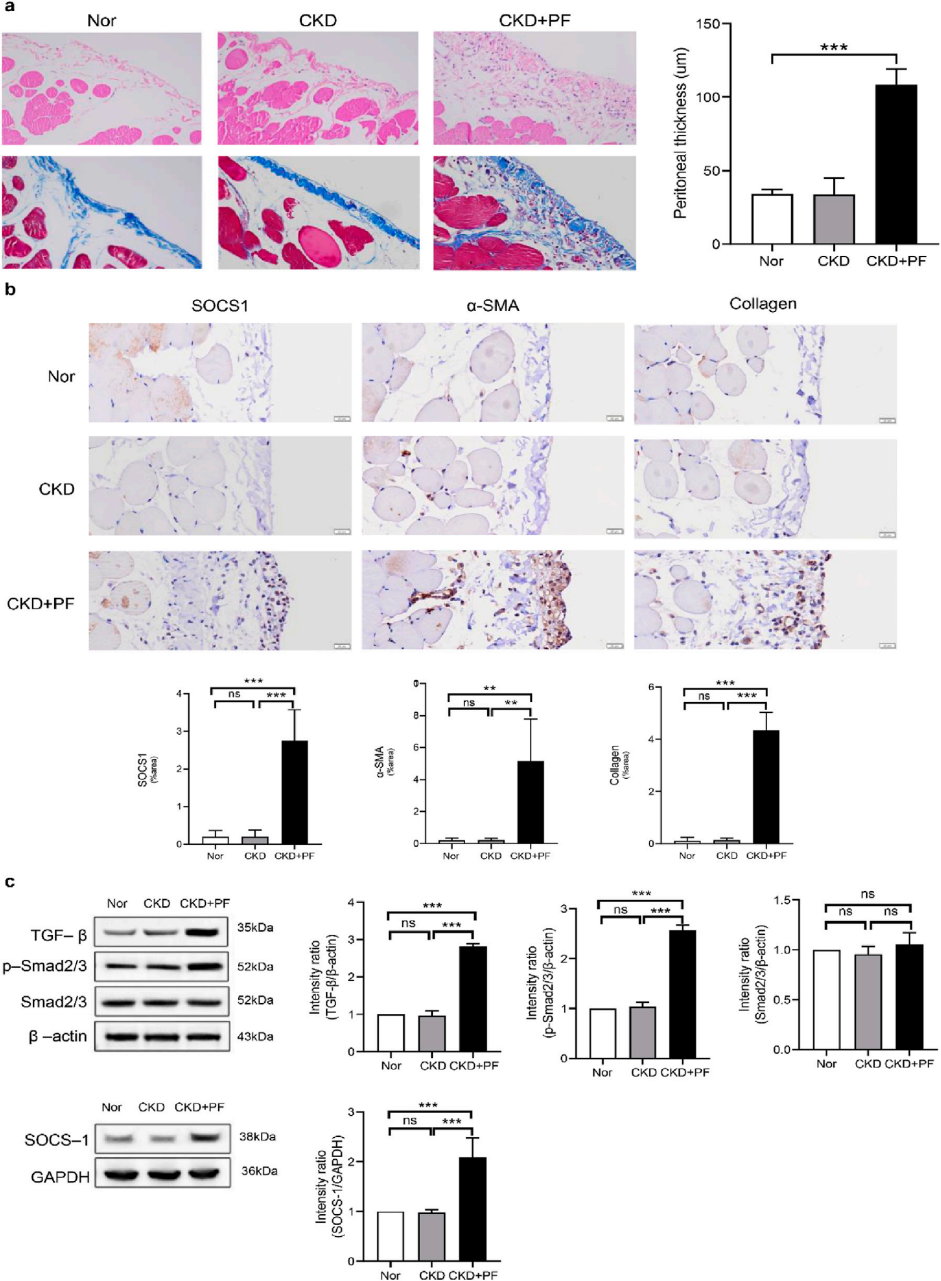
Effect of *SOCS1* on peritoneal fibrosis in rats. **(a)** Masson’s trichrome staining and HE is staining of peritoneal tissue sections (×400). **(b)** Immunohistochemical staining of *SOCS1*, α-SMA, and Collagen I in peritoneal tissue sections (×400). **(c)** Expression levels of TGF-β, Smad2/3, p-Smad2/3, and *SOCS1* were detected using protein blotting. Each group contained eight rats, and three fields of view were randomly selected for each group. The percentage of the stained area was calculated for statistical analysis. All immunoblotting experiments were performed independently and repeated at least three times. Values are expressed as mean ± standard deviation. *p < 0.05 vs. Control; **p < 0.01 vs. Control; ***p < 0.001 vs. Control.

To explore the correlation between *SOCS1* or peritoneal mesothelial cell EMT and fibrosis, we established a combined rat model of CKD with peritoneal fibrosis. As demonstrated in Figure 10, the model has proven to be successful. When compared with the NS group and the CKD group, the PF group demonstrated statistically significant differences in ultrafiltration volume and peritoneal transport function (MTG, D/P, D4/D0). Subsequently, we compared pathological alterations in the peritoneal tissues of different rat groups (Nishimura et al., 2008). Haematoxylin and eosin staining revealed that the peritoneal mesothelial cells of rats in the CKD combined PD group had proliferation of mesenchymal fibrotic tissue, accompanied by inflammatory cell infiltration and a significant increase in neovascularisation, as compared with those of rats in the normal or CKD groups (Figure 11a). Masson’s trichrome staining, along with quantitative analysis, indicated a higher degree of peritoneal fibrosis in the CKD combined PD group than in the normal group. Similarly, immunohistochemical staining showed that the expression levels of mesenchymal markers α-SMA and Collagen I, as well as myofibroblast marker *SOCS1*, were significantly upregulated in the peritoneal tissues of rats in the CKD combined PD group compared with those in the normal or CKD group (Figure 11b). The immunoblotting results were consistent with the immunohistochemical observations. Additionally, the expression of TGF-β1 and p-Smad2/3 was significantly upregulated in the CKD combined PD group of rats compared with the normal and CKD groups of rats, whereas the total Smad2/3 expression did not change significantly among the three groups (Figure 11c). These findings underscore the pivotal involvement of *SOCS1* in peritoneal EMT and the development of fibrosis in rats with CKD and peritoneal fibrosis.

## Discussion

The gold standard for diagnosing peritoneal fibrosis is a peritoneal biopsy, which is invasive. Currently, biomarkers of peritoneal dialysis effluents are used to predict the onset of peritoneal fibrosis. These biomarkers typically include common inflammatory markers. However, reliable biomarkers for assessing peritoneal function and injury are lacking; hence, investigating specific diagnostic markers and infiltrating immune cells could enhance the prognosis of patients undergoing PD. In this study, bioinformatic methods were used to screen for molecular markers and associated pathways in peritoneal fibrosis. The correlation between key genes and immune cells was analysed using CIBERSORT to determine the pattern of immune cell infiltration during disease.

A gene expression dataset related to peritoneal fluid in patients undergoing PD from the GEO database was downloaded, and 121 DEGs were identified under differential expression thresholds. GO, KEGG, and GSEA analyses were performed on the DEGs to analyse the molecular mechanisms leading to peritoneal fibrosis progression. GO analysis revealed that DEGs primarily participate in inflammatory processes, notably in the regulation of leukocyte cell-cell adhesion and positive regulation of T cell activation. KEGG and GSEA analyses emphasised the significance of primary immunodeficiency and Th1 and Th2 cell differentiation pathways in the context of peritoneal fibrosis. In patients who develop peritonitis, the influx of proteases and reactive oxygen species (ROS) secreted by neutrophils induces an initial inflammatory response due to the accumulation of proteases and ROS, and the production of multiple inflammatory factors favours the recruitment and activation of monocytes, which causes the first wave of neutrophils to be subsequently replaced by a monocyte infiltrate (Goodlad et al., 2020). Prolonged dialysate stimulation triggers chronic inflammation of the peritoneum and elevated levels of pro-inflammatory cytokines, which may lead to depletion of immune cell function, similar to the abnormalities of immune tolerance in patients with Primary Immunodeficiency (Goodlad et al., 2020). The composition of lymphocytes in the peritoneal fluid of healthy adults varies with blood lymphocytes, with B-cells accounting for approximately 2.3% of the total body fluid, whereas T cell subsets behave differently (Rajakariar et al., 2008).

To identify the most critical markers, three independent ML algorithms (XGBoost, SVM, and RF) were used to screen the variables. By taking the intersection of the variables screened by the three methods, four DEGs (*SOCS1*, *PIM2*, *HSH2D*, and *MYO3B*) were validated as diagnostic markers for peritoneal fibrosis development in patients undergoing PD using the test set. Higo et al. analysed the expression of 612 kinase-encoding and cancer-associated genes using next-generation sequencing of lung tissues from patients with idiopathic pulmonary fibrosis (IPF) and healthy controls and demonstrated that *PIM2* is upregulated in some cases; thus, they concluded that *PIM2* may serve as a personalised therapeutic target for IPF (Higo et al., 2022). *HSH2D* is a critical regulator of T cell activation and immune responses, as its overexpression inhibits the production of IL-2, a vital cytokine for T cell growth, thereby limiting T cell activity and maintaining immune system balance to prevent autoimmune reactions (Wang and Xiong, 2020).

*SOCS1*, an intracellular protein that suppresses cytokine signalling, participates in a negative feedback loop that attenuates cytokine signalling transduction (From GeneCards). Studies on the role of *SOCS1* in fibrosis are limited. Takafumi et al. found a close correlation between the severity of liver fibrosis and methylation of *SOCS1* in patients with chronic liver disease. Moreover, in the absence of *SOCS1*, mice exhibit more severe liver function damage (Yoshida et al., 2004). Similarly, Taku et al. noted that *SOCS1*-deficient mice are more susceptible to lung infections and pulmonary fibrosis than wild-type mice. Additionally, lower *SOCS1* expression was observed in human lung specimens from patients with IPF than in samples from patients without IPF, indicating the role of *SOCS1* as a pulmonary fibrosis inhibitor (Nakashima et al., 2008). Our study revealed elevated *SOCS1* expression in rats with peritoneal fibrosis, indicating its association with this condition. However, contrary to prior research, *SOCS1* expression is elevated in peritoneal dialysate cells from patients with peritoneal fibrosis. This discrepancy may be attributed to variations in organ involvement, disease stage differences and immune mechanisms.

Given the significance of immune cell infiltration in peritonitis and peritoneal fibrosis among patients undergoing PD, our study used the CIBERSORT algorithm to thoroughly assess immune infiltration in peritoneal fibrosis. This revealed elevated levels of naïve B cells and M0 macrophages, along with decreased monocyte counts in the PD fluid of patients undergoing prolonged PD compared with the control group. Furthermore, correlation analysis revealed a significant negative correlation between *SOCS1* and monocytes; in acute peritonitis models, neutrophils and monocytes were recruited to the inflamed peritoneum. Infiltrating monocytes undergo differentiation into macrophages and dendritic cells, where they localise effector functions. Several studies have demonstrated the importance of peritoneal macrophages in the first line of host peritoneal defence in patients undergoing PD (Liao et al., 2016; 2017). Monocyte chemotactic protein (MCP)-1 plays a role in the pathogenesis of peritoneal fibrosis by affecting the recruitment and activation of monocytes and macrophages. It facilitates the entry of immune cells into the peritoneal cavity, which results in the release of pro-fibrotic cytokines, such as TGF-β1 and fibroblast growth factor. These cytokines are crucial in the development of fibrosis as they promote fibroblast activation and accumulation of the ECM. MCP-1 initiates the inflammatory response and contributes to the progression of peritoneal fibrosis by creating a more fibrogenic local environment (Lee et al., 2012). This demonstrates the importance of monocytes in the development of peritonitis and peritoneal fibrosis in patients undergoing PD.

In patients with ESRD, alterations in the expression of immune cells affect the regulation of immunity and inflammation (Duni et al., 2021). Peripheral blood monocytes in patients with renal failure are activated and involved in the differentiation, cytokine production, and survival of monocytes and lymphocytes (Goodlad et al., 2020). Research has indicated that patients with PD exhibit a decrease in total lymphocyte and B lymphocyte counts in peripheral blood, accompanied by an increase in CD14++CD16^+^ monocyte levels (Duni et al., 2021). Mehdi et al. (Rastmanesh et al., 2009) collected the peripheral blood of undialysed patients with ESRD, haemodialysis patients, patients undergoing PD, and healthy adults and measured their *SOCS1* protein expression and cytokine profiles. They showed that increased monocyte and lymphocyte *SOCS1* expression in patients with ESRD is accompanied by elevated plasma levels of IL-6, TNF-α, and CRP, indicating activation of intracellular inflammatory pathways. In patients undergoing PD, chronic inflammation is a key factor in peritoneal fibrosis; however, no study has explored the correlation between *SOCS1* and peritoneal inflammation or fibrosis in patients undergoing PD. Our findings indicate a correlation between *SOCS1* levels in PD fluid and markers of inflammation in the blood, suggesting that *SOCS1* contributes to peritoneal fibrosis by enhancing the inflammatory immune response.

The TGF-β/Smad signalling pathway, a key mediator in the pathogenesis of peritoneal fibrosis, is activated in patients undergoing continuous ambulatory PD, particularly in the presence of peritoneal tissue thickening (Duan et al., 2014). Additionally, chronic exposure to peritoneal dialysate triggers significant peritoneal fibrosis, whereas Smad3 knockout prevents peritoneal fibrosis, in wild-type mice (Giri et al., 2023). Mesothelial-to-mesenchymal transition is an important mechanism of peritoneal fibrosis resulting from long-term PD (Aroeira et al., 2007). α-SMA and E-cadherin serve as markers indicating the transdifferentiation of cells into myofibroblasts (Liu et al., 2008). TGF-β1 can decrease the gene and protein expression of E-cadherin while upregulating the expression of α-SMA and Collagen I at the gene and protein levels. This implies a weakening of intercellular adhesion and an increase in cell motility and migration, as well as a transformation of cells into myofibroblasts, ultimately leading to the overexpression of the ECM and fibrosis (Yao et al., 2008). Our results suggest that *SOCS1* plays a key role in the onset and development of peritoneal EMT and fibrosis in rats with CKD and peritoneal fibrosis. The inhibition of *SOCS1* expression may attenuate peritoneal EMT and fibrosis by modulating the TGF-β/Smad pathway in CKD and peritoneal fibrosis rats. In the future, we plan to investigate the relationship between *SOCS1* and the TGF-β/Smad pathway in depth. Through clinical experiments, we found that the levels of *SOCS1* and TGF-β1 in the peritoneal effluent of patients undergoing PD were closely related to the duration of dialysis. After more than 16 months of PD treatment, the expression level of TGF-β1 in the peritoneal effluent increased significantly with increased dialysis duration. The level of *SOCS1* in the PD effluent increased significantly with increased dialysis duration after more than 21 months and was consistent with the TGF-β1 trend.

We innovatively used multiple ML methods to screen and validate diagnostic markers for the development of peritoneal fibrosis in patients undergoing PD using the CIBERSORT method to detect peripheral blood immune cell infiltration. However, this study had some limitations. First, our analysis involved secondary mining of a previously published dataset. Consequently, the conclusions drawn may differ owing to the application of different analytical ideas and perspectives. The CIBERSORT algorithm is based only on limited transcriptomic data, and the distribution of some low-abundance immune cell subpopulations in patients with peritoneal fibrosis remains incompletely characterised. Second, we used only one dataset because there is a lack of relevant datasets for peritoneal dialysate; thus, it was validated using its own clinical samples, but the number of cases was small. Larger clinical studies are needed in the future to validate the results and explore the mechanisms in further detail. Third, in animal experiments, no specific mechanism was explored; future research should overexpress or inhibit the expression of *SOCS1* to clarify the specific signal transduction pathway in which *SOCS1* acts. Finally, this study did not validate or explore the relationship and mechanism of action of *SOCS1* with immune cell infiltration in animal or clinical studies.

## Conclusion

Bioinformatics analysis and ML algorithms validated *SOCS1* as a potential marker of peritoneal fibrosis progression in patients undergoing PD. Monocytes may play a role in the fibrotic process and are closely associated with *SOCS1* expression. Therefore, this study offers a novel perspective and approach for the diagnosis and treatment of peritoneal fibrosis, particularly from an immunoregulatory standpoint.

## Data Availability

The original contributions presented in the study are included in the article/supplementary material, further inquiries can be directed to the corresponding authors.
